# Bi-Directional Exchange of Membrane Components Occurs during Co-Culture of Mesenchymal Stem Cells and Nucleus Pulposus Cells

**DOI:** 10.1371/journal.pone.0033739

**Published:** 2012-03-15

**Authors:** Sandra Strassburg, Nigel W. Hodson, Patrick I. Hill, Stephen M. Richardson, Judith A. Hoyland

**Affiliations:** 1 Regenerative Medicine, School of Biomedicine, Faculty of Medical and Human Sciences, The University of Manchester, Manchester, United Kingdom; 2 School of Chemical Engineering, Faculty of Engineering and Physical Sciences, The University of Manchester, Manchester, United Kingdom; University of Sao Paulo – USP, Brazil

## Abstract

Mesenchymal stem cell (MSC)-based therapies have been proposed as novel treatments for intervertebral disc (IVD) degeneration. We have previously demonstrated that when MSCs are co-cultured with nucleus pulposus (NP) cells with direct cell-cell contact, they differentiate along the NP lineage and simultaneously stimulate the degenerate NP cell population to regain a normal (non-degenerate) phenotype, an effect which requires cell-cell communication. However, the mechanisms by which NP cells and MSCs interact in this system are currently unclear. Thus, in this study we investigated a range of potential mechanisms for exchange of cellular components or information that may direct these changes, including cell fusion, gap-junctional communication and exchange of membrane components by direct transfer or via microvesicle formation. Flow cytometry of fluorescently labeled MSCs and NP cells revealed evidence of some cell fusion and formation of gapjunctions, although at the three timepoints studied these phenomena were detectable only in a small proportion of cells. While these mechanisms may play a role in cell-cell communication, the data suggests they are not the predominant mechanism of interaction. However, flow cytometry of fluorescently dual-labeled cells showed that extensive bi-directional transfer of membrane components is operational during direct co-culture of MSCs and NP cells. Furthermore, there was also evidence for secretion and internalization of membrane-bound microvesicles by both cell types. Thus, this study highlights bi-directional intercellular transfer of membrane components as a possible mechanism of cellular communication between MSC and NP cells.

## Introduction

A change in cellular phenotype of the nucleus pulposus (NP) cells residing in the inner core of the intervertebral disc (IVD), leading to increased extracellular matrix degradation and altered matrix synthesis, is considered to be one of the major causes of IVD degeneration which is strongly associated with low back pain [1]. Traditional therapies for IVD degeneration are mainly restricted to those that treat the pain and do not target the underlying aberrant cell biology. However, with the advent of tissue engineering and regenerative medicine, novel cell-based therapies are being investigated with the ultimate aim of replacing NP cells and repairing the degenerate IVD [2]. Since autologous and/or allogeneic NP cells are not an ideal cell population, mesenchymal stem cells (MSCs) have been proposed as the preferred cell source for IVD regeneration [3], [4].

MSCs can be easily isolated from a number of sources including bone marrow, rapidly expanded and differentiated along several mesenchymal lineages *in vitro* including differentiation to NP-like cells [5], [6], [7]. Additionally, *in vivo* studies have shown that implantation of MSCs into experimentally induced degenerate animal discs leads to restoration of disc structure in terms of improved IVD height and accumulation of proteoglycans [8], [9], [10], [11], [12], [13]. However, the exact mechanism by which this regeneration occurs is not fully understood. Once implanted, MSCs are able to interact with the surrounding microenvironment and as such a variety of mechanisms by which MSCs might exert their biological effects have been postulated, including replacement of lost/degenerate cells through differentiation of MSCs into functional NP cells or provision of trophic support/stimulation for the native NP cells.

In order to ascertain the mechanism of action, several investigators have utilised *in vitro* co-culture model systems to address the question whether MSCs differentiate to an NP-like phenotype or whether MSCs have a stimulatory effect on native NP cells [7], [14], [15], [16]. These studies have yielded varying results depending on the nature of the co-culture system employed (monolayer, 3D, indirect or direct co-culture). We have previously demonstrated, using a direct and an indirect co-culture system of MSCs and NP cells, that direct cell-to-cell contact is essential for MSC differentiation to an NP-like phenotype as characterized by increases in matrix-associated NP marker genes [14]. Furthermore, we have shown using this direct co-culture model system, that MSCs only have stimulatory effects on NP cells that are derived from degenerate discs and not on those derived from non-degenerate discs [7]. Thus, therapeutic effects of stem cell therapy may not be solely due to replacing lost/degenerate NP cells with MSCs but may also be due to paracrine mechanisms or cell-to-cell interactions leading to MSC differentiation and an altered native NP phenotype. However, the nature of such NP-to-MSC interactions is not fully understood.

Evidence from different research areas have indicated that cell-to-cell communication directing stem cell differentiation can be regulated by intercellular transfer of cellular components, through mechanisms such as cell fusion [17], [18], [19], gap-junctional communication [20] and exchange of membranous components via microvesicles [21], [22]. This includes other musculoskeletal cells, including articular cartilage chondrocytes and tendon cells [23], [24]. Importantly, all of these mechanisms may be physiological phenomena which can transfer soluble, cellular or nuclear components, including functional genes between cells, ultimately causing phenotypic alterations.

However, to date, there is no evidence to support the hypothesis of bi-directional intercellular transfer of cellular components between MSCs and NP cells during co-culture with direct cell-to-cell contact. As all of these events have been previously reported to affect the phenotype of target cells, we investigated whether cell fusion or transfer of cytoplasm or membranous components may be operational during direct co-culture of MSCs and NP cells and thus may be responsible for the previously reported MSC differentiation toward NP cells and improvement of degenerate NP cell phenotype. Our data established a minor role for cell fusion and gap-junctional communication and interestingly identified extensive bi-directional membrane transfer between MSCs and NP cells as the major mechanism by which cellular components are transferred.

## Materials and Methods

### Ethics statement

MSCs were isolated from bone marrow obtained during either total hip or knee replacement following approval from the North West Research Ethics Committee and fully informed written consent of patients. Intervertebral disc tissue was obtained with fully informed written consent and North West Research Ethics Committee approval from patients undergoing discectomy.

### Cell culture

Human MSCs (3 female, 3 male, mean age: 72 years; age range 60–85 years) were obtained using established methodology [7]. Briefly, bone marrow aspirates were washed with phosphate buffered saline (PBS, PAA Laboratories), incubated with RosetteSep (StemCell Technologies Inc) and layered on Histopaque-1077 (Sigma) for gradient centrifugation. Adherent mononuclear cells were cultured in Minimum Essential Medium, α-modification (α-MEM, Gibco) and used at passage 3 for all experiments. The multipotentiality of MSCs was assessed via differentiation along the three common mesenchymal lineages (osteogenic, adipogeneic and chondrogenic) (data not shown) using standard methodology [25].

NP cells (3 female, 1 male, mean age: 44 years; age range 39–51 years) were obtained from surgical degenerate lumbar (histological grade 7–11 [26]; L4/5-L5/S1) IVD tissue using established methodology [7]. Briefly, NP tissue was enzymatically digested and cells cultured in Dulbecco's Modified Eagle Medium (DMEM, Gibco).

All cell cultures were maintained in a laminar flow hood class-II and incubated at 37°C with 5% CO_2_ and 20% oxygen in a humidified environment with a media change 2–3 times a week.

### Co-cultivation of fluorescently labeled MSCs and NP cells

For all cell labeling, 1×10^6^ cells were resuspended in 1 ml Hank's Buffered Phosphate Saline (HBSS, PAA Laboratories) and incubated with the appropriate dye (see following sections) for 30 minutes at 37°C in the dark followed by two wash steps in medium. Direct co-cultures of labeled MSCs and NP cells were performed in monolayer at 50∶50 ratios in 6-well plates (Becton Dickinson) as described previously [7], [14]. Labeled MSC or NP cells in monocultures alone served as controls. Co-cultures and controls were cultivated in DMEM with 10% FCS for specified time intervals.

### Assessment of cell fusion

Cellular fusion was ascertained by 5,6 carboxyfluorescein diactetate, succinimidyl ester (CFDA; Invitrogen) and SNARF-1 carboxylic acid, acetate, succinimidyl ester (SNARF-1; (Invitrogen). Prior to direct co-culture, MSCs were fluorescently labeled with a final concentration of 10 µM CFDA and NP cells were labeled with 10 µM SNARF-1. After 1, 3 and 7 days, all co-cultures and controls were analysed for CFDA and SNARF-1 fluorescence using flow cytometry. Cellular fusion was demonstrated by double labeled cells. Data was obtained from two different experiments. To further characterize cellular fusion, CFDA and SNARF positive cells were sorted (BD Biosciences FACS Aria high speed cell sorter with Diva 5 software) as previously described [7]. Cells were then washed and centrifuged onto a microscope slide and fluorescence microscopy used to confirm that double labeled cells were generated by cell fusion and not by random cell aggregation.

### Assessment of gap-junctions

Transmission electron microscopy (TEM) was used to visualize gap-junctions at the site of MSC-to-NP cell contact. CFDA-labeled MSCs and SNARF-labelled NP cells were directly co-cultured on a gridded coverslip (MatTek). Initially, fluorescence microscopy was used to identify sites of cellular contact between MSCs and NP cells and the grid on the coverslip illustrated the position of cells for subsequent TEM analysis.

Subsequently gap-junctional communication was investigated using calcein- AM (calcein; Invitrogen) and Vybrant CM-DiL cell-labeling solution (DiL; Invitrogen).

Donor cells (MSC or NP cells, respectively) were double labeled with 5 µM DiL and µM calcein and co-cultured with unlabeled recipient cells (NP cells or MSCs, respectively). After 24 hours, functional gap-junctions were assessed by flow cytometry. When gap-junctions are established, cytosolic calcein transfer from donor to recipient cells occurs and initially unlabeled recipient cells exhibit the green fluorescence of calcein but not the red fluorescence of DiL.

### Assessment of transfer of membrane components

Transfer of membrane components between cells in co-culture was assessed by the lipophilic dye DiL. Prior to co-culture, donor cells (MSC or NP cells, respectively) were double labeled with 5 µM DiL and 10 µM CFDA and co-cultured with unlabeled recipient cells (NP cells or MSC respectively). After 1, 3 and 7 days, all co-cultures and controls were analyzed using flow cytometry. Transfer of membrane components was quantified by DiL transfer from donor to unlabeled recipient cells, meaning initially unlabeled recipient cells exhibited the red fluorescence of DiL, but not the green fluorescence of CFDA.

### Flow cytometry analysis

Analysis of cellular fluorescence was performed using a Cyan flow cytometer with Summit V4.3 software. Cells were trypsinized and washed in HBSS to remove all remaining media and serum components and resuspended in 400 µl HBSS. Cells pass a 488 nm laser beam and were first analyzed in a 2D dot plot for cell size and granularity by forward and side scatter. Vital cells were gated and further analyzed by pulse width to exclude cell aggregates. Single cells were analyzed for fluorescence beyond 530 nm.

### Isolation of microvesicles

Microvesicles (MVs) were isolated from media used during direct co-culture of MSCs with NP cells. After centrifugation at 2000 g for 10 minutes to remove cell debris, cell free supernatants were ultra-centrifuged (Optima TL-100, Beckman Coulter) at 100000 g for 1 hour at 4°C in polycarbonate centrifugation tubes; the resulting MV-pellet was washed in HBSS and submitted to a second ultracentrifugation step.

### Imaging of microvesicles - scanning electron microscopy

For scanning electron microscopy (SEM), isolated microvesicles were fixed in 100 µl glutaraldehyde, dehydrated in ascending alcohol solutions, dried on a glass coverslip and sputter coated with gold using an SC500 coating unit. The specimens were then imaged using a scanning electron microscope (FEI Quanta 200) with an accelerating voltage of 15–30 keV and at a working distance of between 6 and 10 mm.

### Cellular incorporation of MVs

The secretion of DiL-labeled MVs was induced by direct co-culture with one DiL-labeled cell population (either MSCs or NP cells, respectively) for 7 days. Due to necessary medium changes, the conditioned media from these co-cultures were taken at day 3 and 7 and MVs isolated by ultracentrifugation. Isolated MVs may have been MSC or NP cell derived, but subsequent analysis identified DiL-labeled MV uptake by unlabelled cells only. DiL-labeled MVs from one cell population were transferred onto unlabeled cells of the other cell type and incubated for 24 hours. Additionally, whole conditioned medium and supernatant of the MV-pellet after ultracentrifugation was transferred onto unlabeled cells as a control that DiL is specifically bound to MVs. Flow cytometry was used to identify DiL fluorescence within the unlabeled cell population indicative of MV incorporation into cells.

## Results

### Cell fusion between MSC and NP cells

**Figure 1:**
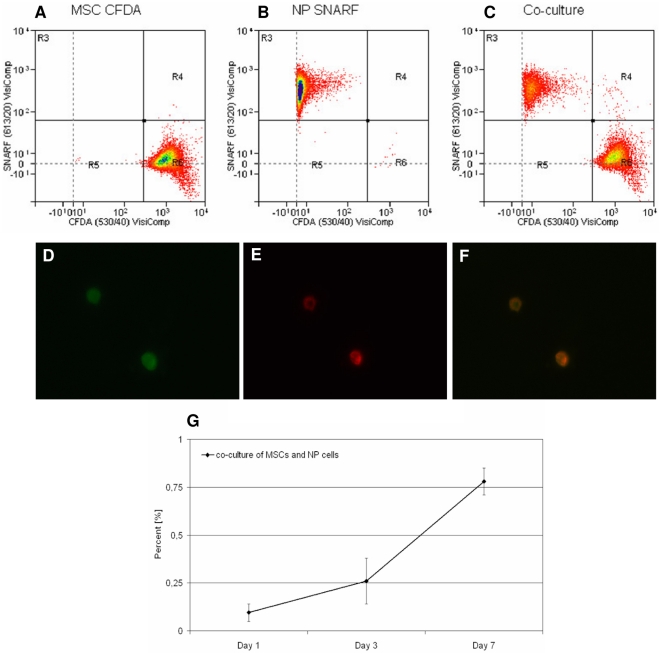
Cell fusion of MSCs and NP cells during direct co-culture. A–C: An exemplar flow cytometry analysis for CFDA labeled MSCs and SNARF labeled NP cells after 7 days: A) CFDA labeled MSCs alone; B) SNARF labeled NP cells alone; C) Co-culture of CFDA labeled MSCs and SNARF labeled NP cells. D–F: Fluorescence microscopy of sorted double labeled cells from region R4: D) Cells labeled with CFDA; E) Cells labeled with SNARF; F) Color combine; enlargement of a 100× magnification. G) Percentage of cell fusion after 1, 3 and 7 days calculated from flow cytometry data. Error bars indicate standard error of the mean. Abbreviations: MSC: mesenchymal stem cell; NP: nucleus pulposus; CFDA: 5,6 caboxyfluorescein diactetae, succinimidyl ester; SNARF: carboxylic acid, acetate, SE.

Cellular fusion between MSCs and NP cells was assessed by flow cytometry after 1, 3 and 7 days of direct co-culture (Figure 1A–C). CFDA labeled MSCs and SNARF labeled NP cells were either analyzed alone or together in direct co-culture. CFDA labeled MSCs appeared in region R6, SNARF labeled NP cells in region R3 and double labeled cells in region R4, suggesting that these cells were a result of cell fusion between MSCs and NP cells.

To confirm that these double labeled cells had arisen by cell fusion and not by aggregation, double labeled cells were sorted and analyzed by microscopy (Figure 1D–F) which showed that double labeled cells had fused from MSCs and NP cells during direct co-culture. These double labeled cells represented single cells that were CFDA positive (as former MSCs) as well as SNARF positive (as former NP cells).

Analysis of the flow cytometry data revealed that in a co-culture of MSCs and NP cells, the percentages of double labeled cells in region R4 increased from 0.1% at day 1 to 0.26% at day 3 and to 0.78% at day 7 (Figure 1G).

### Gap-junctional communication between MSCs and NP cells

**Figure 2:**
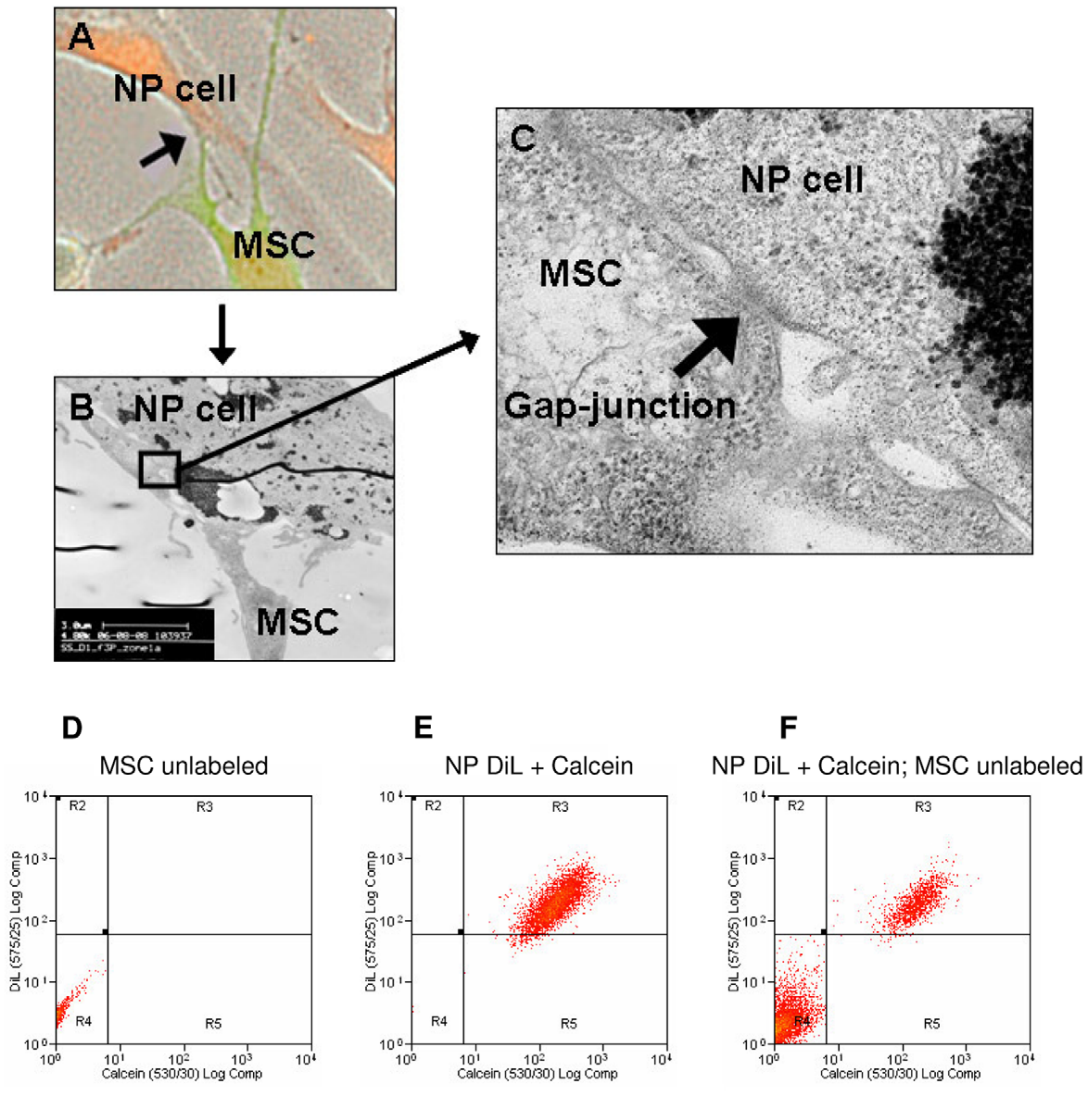
Formation of gap-junctions. Example images of gap-junctions: A) Fluorescence microscopy to illustrate a potential site of cell-to-cell contact (arrow) between MSCs (green) and NP cells (red). B) TEM of the site of cellular contact between the MSC and NP cell identified in panel A. C) Enlargement of area (blocked in panel B) depicting cell-to-cell contact between MSC and NP cell revealing a typical gap-junctional structure (arrow). Example flow cytometry dot plots to identify gap-junctional dependent dye transfer between MSCs and NP cells: D) Unlabeled MSCs. E) DiL and calcein labeled NP cells. F) Direct co-culture of unlabeled MSCs and double labeled NP cells after 24 hours. No calcein only labeled cells were detectable. Abbreviations: MSC: mesenchymal stem cell; NP: nucleus pulposus.

TEM studies suggested the presence of gap-junctions forming between MSCs and NP cells during direct co-culture (Figure 2A–C).Fluorescence microscopy of CFDA-labeled MSC (green) and SNARF-labeled NP cells (red) on a gridded coverslip identified the site of potential cellular contact (Figure 2A). Subsequently, TEM identified structures resembling gap-junctions at this site of MSC-to-NP cell contact (Figures 2B and 2C). Therefore, in an attempt to demonstrate functional gap-junctions, a calcein-transfer assay was performed on co-cultures over a period of 24 hours (Figure 2D–F). Unlabeled MSCs alone in region R4 (Figure 2D) and DiL/calcein-labeled NP cells alone in the region R3 (Figure 2E) were co-cultured with direct cell-to-cell contact and if there was cytosol transfer through functional gap-junctions, cells labeled with calcein-only should be visible in region R5. Flow cytometry data did not show significant numbers of calcein positive cells after 24 hours of direct co-culture. No calcein dye transfer from double labeled NP cells to unlabeled MSCs was observed (Figure 2F) or when the same experiment was conducted in the reverse direction (MSCs to NP cells).

### Membrane transfer between MSCs and NP cells

**Figure 3:**
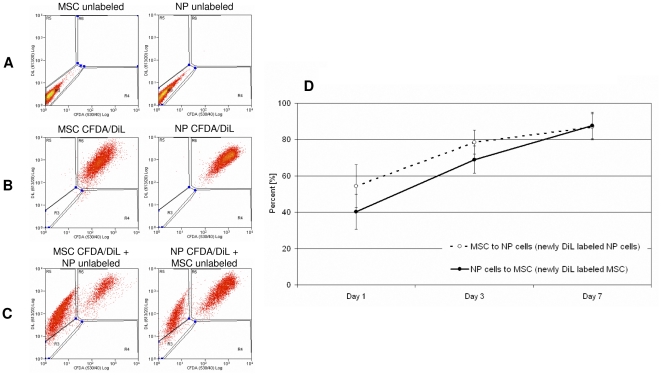
Transfer of membrane components during direct co-culture of MSC and NP cells. A–C: Exemplar flow cytometry to quantify transfer of membrane (DiL) components after 7 days. A) Dot plots for unlabeled MSCs and NP cells. B) Dot plots for CFDA and DiL labeled MSCs and NP cells. C) Dot plots for CFDA and DiL double labeled MSCs co-cultured with unlabeled NP cells; CFDA and DiL double labeled NP cells co-cultured with unlabeled MSCs. D) Percentages of DiL transfer after 1, 3 and 7 days from a labeled cell to an unlabeled cell during direct co-culture calculated from flow cytometry data. Error bars indicate standard error of the mean. Abbreviations: MSC: mesenchymal stem cell; NP: nucleus pulposus; CFDA: 5,6 carboxyfluorescein diactetae, succinimidyl ester; DiL: Vybrant CM-DiL cell-labeling solution.

The transfer of membrane components between MSCs and NP cells is shown in Figure 3 which shows exemplar flow cytometry data for unlabeled MSCs and NP cells in region R3 (3A) and CFDA and DiL double labeled MSCs and NP cells in region R6 (3B). These cells were co-cultured with direct cell-to-cell contact for 7 days. Cell cytometry analysis revealed DiL dye transfer from a labeled cell to an unlabeled cell, demonstrated by DiL-only labeled cells in region R5 (3C).

Analysis of the flow cytometry data showed that both MSCs as well as NP cells were able to transfer membrane components to the other cell population during direct co-culture without significant differences between directions of transfer. DiL transfer from labeled cells to unlabeled cells increased over time and 7 days after direct co-culture 87.0% of former unlabeled NP cells were positively labeled with membrane components derived from MSCs and 87.8% of former unlabeled MSCs were positively labeled with DiL derived from NP cells (Figure 3D).

### MSCs and NP cells secrete microvesicles during direct co-culture

**Figure 4:**
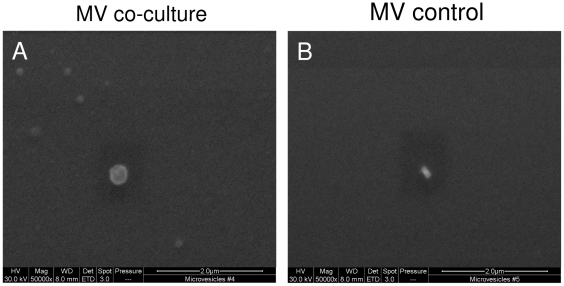
Electron microscopy images of microvesicles. SEM pictures of ultracentrifuged conditioned media derived from a co-culture (A). Pellet demonstrates numerous MVs, which vary in size, but were less than 1 µm. No MVs could be observed in control medium (B). Abbreviation: MV: microvesicle.

One possible mechanism to transfer or exchange membrane components during direct co-culture is via the formation and release of microvesicles. Here, it was hypothesized that microvesicles (from either cell type) were only shed into the medium during direct co-culture. MVs were isolated from media of direct co-cultures and analyzed by SEM. SEM analysis demonstrated round structures typically resembling MVs (appropriate size range of 30 nm-1 µm) (Figure 4A). No such structures were observed in control media samples (Figure 4B).

### Cellular incorporation of microvesicles during direct co-culture

**Figure 5:**
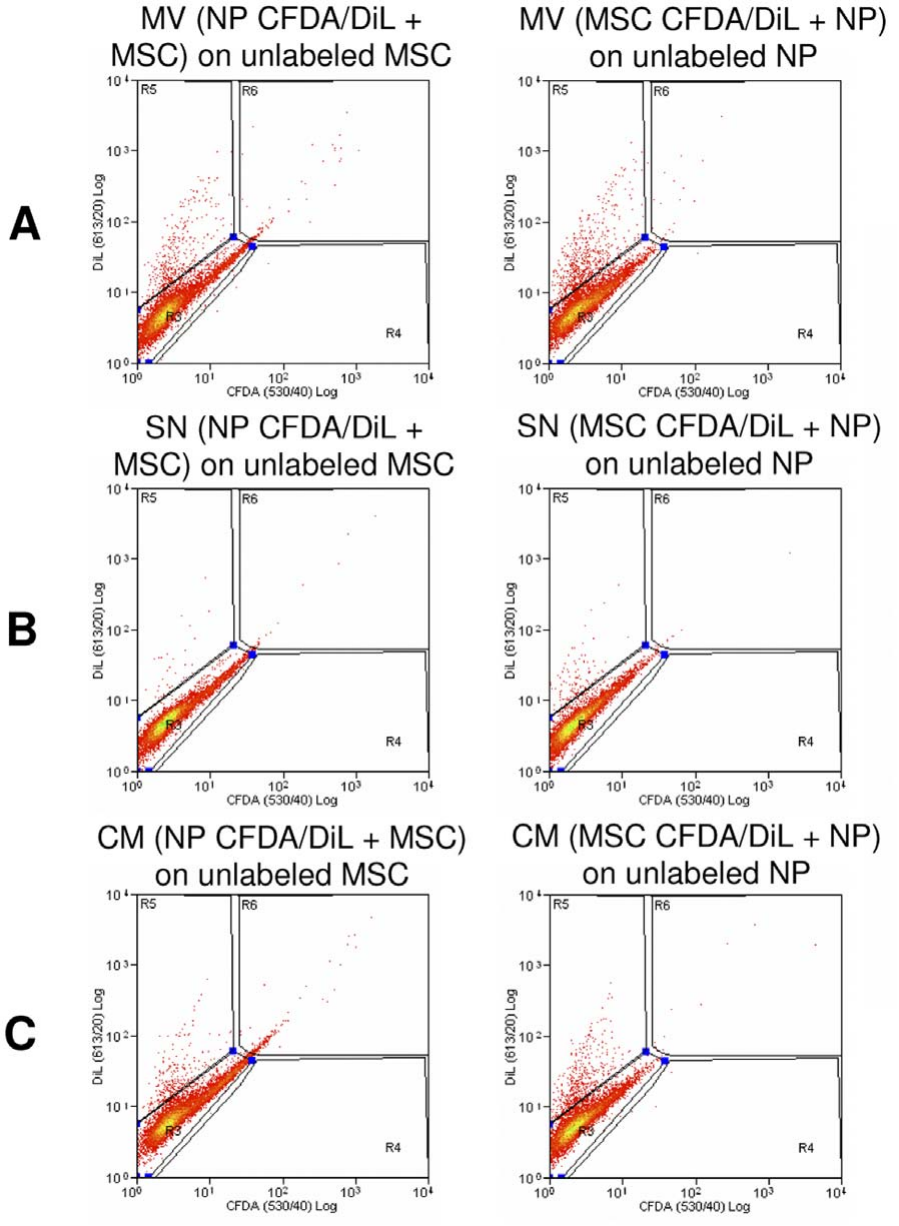
Incorporation of DiL-labeled microvesicles into MSC and NP cells during direct co-culture. Exemplar flow cytometry analysis. A) MVs derived from a direct co-culture of MSCs and NP cells (one cell population was DiL labeled) and incubated with the unlabeled cell population. B) MV-free supernatant after ultracentrifugation. C) Conditioned medium derived from a direct co-culture of MSCs and NP cells (one cell population was DiL labeled). Abbreviations: MSC: mesenchymal stem cell; NP: nucleus pulposus; MV: microvesicles; SN: supernatant; CM: conditioned medium; CFDA: 5,6 caboxyfluorescein diactetae, succinimidyl ester; DiL: Vybrant CM-DiL cell-labeling solution.

Flow cytometry was performed to investigate the incorporation of MSC-derived DiL labeled MVs into the membrane of NP cells or NP derived DiL labeled MVs into MSCs. Figure 5 shows an example plot illustrating the incorporation of DiL labeled MVs into unlabeled cells (5A) which was compared to cells cultured in the MV free supernatant after ultracentrifugation (5B) and whole conditioned medium (5C) derived from a co-culture. 5.67% of MSCs were positive for DiL obtained by DiL positive MVs shed from NP cells and 8.50% of NP cells were positive for DiL obtained from DiL positive MVs shed from MSCs. The supernatant obtained after ultracentrifugation for MVs did not label cells (MSCs 0.68% positive; NP cells 1.32% positive), demonstrating that there is no unspecific dye uptake of “free” dye in the medium and that it was bound to pelleted MVs. Conditioned medium (containing MVs) obtained from a co-culture was effective in labeling unlabeled cells with DiL to the same extent as MVs alone (MSCs 3.59% positive; NP cells 8.15% positive).

## Discussion

Over the last decade, MSCs have been considered as a suitable cell population for replacing or repairing degenerate or injured tissues. IVD degeneration is marked by progressive changes in NP cell phenotype and NP extracellular matrix as a result of increased matrix degradation and altered synthesis. MSC transplantation has been shown to improve outcome in animal models of IVD degeneration in that they restore the normal disc structure and phenotype [8], [10], [11], [12]. However, the mechanisms regulating either MSC differentiation to NP cells or stimulation of NP cells by MSCs are not fully understood. Therefore, we investigated the nature of cellular interactions between MSCs and degenerate NP cells during direct co-culture with emphasis on bi-directional intercellular exchange of membrane/cellular components. We show that transfer of membrane components, and not the often proposed mechanisms of cell fusion or gap-junctional communication, is the primary mechanism of cellular communication between MSCs and NP cells during direct co-culture.

Spontaneous cell fusion is the often proposed mechanism to explain adult stem cell plasticity both in *in vivo* and *in vitro* co-cultures. *In vivo*, the phenomena of cell fusion have been observed with hepatocytes in the liver, cardiomyocytes in the heart and purkinje cells in the brain [17]. *In vitro*, it has been shown that MSCs undergo spontaneous cell fusion with ESCs [19] and heat shock treated small airway epithelial cells [18]. All studies illustrating cell fusion report an altered phenotype of MSCs to that of the host tissue or co-cultured cells, concluding that the altered phenotype of MSCs does not arise by direct conversion to the other cell type but rather through the generation of hybrid cells. Within our direct monolayer co-culture model system, we were able to show that fusion between MSCs and NP cells does occur *in vitro*, although importantly the number of fusion events is rare (less than 1% by day 7). In this respect, these results are similar to those obtained by Vadala et al. [15] who reported 0.2% cell fusion in a 3D pellet co-culture of MSC and NP cells. Thus, it is unlikely that cell fusion is the mechanism behind the MSC differentiation to NP cells and redifferentiation of degenerate NP cells to normal NP cells described previously in this system [7].

Another often proposed mechanism to explain differentiation of MSCs in close proximity to other cells is gap-junctional communication with neighboring cells. Most of the evidence for gap-junctional communication in regards to MSC biology has been described in co-cultures of MSCs and cardiomyocytes. For example, gap-junctions were formed along regions of contact between MSCs and cardiomyocytes characterized by calcein transfer from cardiomyocytes to MSCs and the expression of the gap-junctional protein connexin-43 within 24 hours of direct co-culture [27]. Furthermore, Yoon et al. has demonstrated that MSCs express cardiac markers only after direct co-culture that is characterized by calcein transfer highlighting the presence of gap-junctional communication and not after indirect co-culture or under the influence of neonatal cardiomyocyte-conditioned medium [20]. Here, although morphological examination by TEM revealed structures resembling gap-junctions at the MSC-to-NP cell connection site, there was no evidence of calcein transfer through functional gap-junctions and no immunopositivity for the gap-junctional protein connexin-43 along MSC/NP cell connection sites (data not shown) within the system. As gap junction formation is a transient event it may therefore be more common than our results suggest, given the snapshot nature of the methodology employed. However, the lack of calcein dye transfer between cells in this system suggests that gap-junctional communication does not play a central role in directing MSC differentiation or degenerate NP cell redifferentiation during co-culture.

Membrane transfer during direct co-culture is a possible procedure for cellular communication between MSCs and NP cells. Cell membranes contain varying amounts of lipids and proteins which are involved in a variety of cellular processes including cell signaling. Membrane transfer between MSCs and renal tubular cells in co-culture has been recently established by the fluorescent dye DiO and/or DiD and flow cytometry analysis and/or fluorescence microscopy [28], [29]. Niu et al. reported intercellular transfer of a variety of membrane lipids and transmembrane proteins during cell-cell contact by transient local membrane fusion allowing molecules to migrate by lateral diffusion to adjacent cells [30]. In the current study, after direct co-culture with one DiL-labeled cell type, many of former unlabeled cells demonstrated DiL fluorescence and the number of fluorescently labeled cells increased over time. The substantial DiL transfer of up to 87% to the unlabeled cell population after 7 days of direct co-culture implies that both MSCs and NP cells are able to transfer and to incorporate DiL-labeled membrane components in a bi-directional manner. While this methodology illustrates the transfer of lipid components, it has been hypothesized that membrane proteins exchange at the same time, although the efficiency of dye transfer and thus lipid transfer is shown to be higher [30]. Thus, transfer of membranous components might be essential for MSC differentiation to NP cells as well as for degenerate NP cells reprogramming by adjacent not fully differentiated MSCs.

Membrane transfer can also occur via microvesicles which are membrane derived vesicles of 30 nm-1 µm released into the extracellular environment by a variety of cell types. MVs can interact with different target cells, altering their phenotype toward the MV-releasing cell by delivering host specific molecules, such as lipids, proteins or nucleic acids including mRNA and miRNA (reviewed in [31], [32], [33], [34], [35]).

Here, we successfully demonstrated by SEM the presence of MVs shed into the medium following direct co-culture. We also demonstrated that DiL-labeled MVs derived from a direct co-culture fused with both unlabeled MSCs and NP cells, transferring their fluorescence. Both MSCs and NP cells internalize DiL-labeled MVs from NP cells or MSCs, respectively, demonstrating that membrane transfer by MVs is bi-directional. Thus, the transfer of lipid-, protein- and RNA-containing MVs during direct co-culture between MSC and NP cells might be the underlying mechanism of the formerly observed changes in cell phenotype. However, more detailed studies would be required to identify whether mRNAs or miRNAs may be present in these MVs or whether membrane components may be responsible for affecting cell phenotype.

Although we could identify MVs as a possible mechanism of membrane transfer, our results suggest that transfer of membrane bound MVs is probably not the main mechanism of membrane transfer, since they only count for about 8% newly DiL-labeled cells whereas up to nearly 90% newly DiL-labeled cells could be observed during direct co-culture. Thus, it is assumed that other mechanisms of bi-directional membrane transfer may exist, such as tunneling nanotubes [36] or simply transient membrane fusion and further studies are required to investigate this possibility.

The consequences of membrane transfer between MSCs and NP cells during direct co-culture are important for clinical application of MSCs for IVD regeneration in that MSCs adopt the phenotype of NP cells and degenerate NP cells regain their normal phenotype. Our identification of bi-directional membrane transfer between MSC and degenerate NP cells would be a potential mechanism by which MSCs and NP cells communicate with each other and induce phenotypic changes.
